# Identification and mechanism of key genes in inflammatory response of chronic otitis media in rats

**DOI:** 10.1038/s41598-025-24626-z

**Published:** 2025-11-19

**Authors:** Oulai Li, Yan Lin, Rui Wang, Jiazhi Yin, Lijuan Zhou, Yong Tang

**Affiliations:** 1 Department of Otolaryngology, Head and Neck Surgery, The First Affiliated Hospital of Kunming Medical University, Kunming, 650000 China; 2 Department of Otolaryngology Head and Neck Surgery, The Affiliated Children’s Hospital of Kunming Medical University, Kunming, 650000 China

**Keywords:** Chronic suppurative otitis media, Inflammatory response, Key genes, Transcriptome sequencing, Molecular docking, Immunohistochemical analysis, Diseases, Medical research, Pathogenesis

## Abstract

Chronic suppurative otitis media (CSOM) affects 330 million people globally; however, biomarkers for its inflammatory response (IR) remain underinvestigated. This study aimed to identify key genes in IR and explore the mechanisms of CSOM. Following the CSOM model construction, RNA sequencing was performed on rat samples and IR-related genes were extracted from public databases. The key genes were identified using the support vector machine recursive feature elimination and least absolute shrinkage and selection operator algorithms, and the diagnostic performance was assessed using receiver operating characteristic curves. Nucleoside diphosphate kinase (NDK) protein expression was specifically validated via immunohistochemical analysis. A nomogram was created to predict the incidence of CSOM, and functional enrichment, drug prediction, and molecular docking analyses were conducted. The results indicated that three key genes (*Has2*, *Clec5a*, and *Il6*) were markedly upregulated in CSOM, validated by immunohistochemical staining and quantitative polymerase chain reaction analysis. These findings provide insights into potential CSOM therapies.

**Supplementary Information:**

The online version contains supplementary material available at 10.1038/s41598-025-24626-z.

## Introduction

Chronic suppurative otitis media (CSOM) is a prevalent and clinically relevant ear disorder. It is characterized by a sustained infection within the middle ear, and its hallmark feature is a perforated tympanic membrane. It indicates a chronic inflammatory process affecting the middle ear and mastoid cavity. The hallmark presentation of CSOM is chronic or persistent ear discharge through a perforated tympanic membrane, which lasts more than 2 to 6 weeks. CSOM can be broadly classified into CSOM with and without cholesteatoma, each presenting different pathophysiological mechanisms and clinical management approaches^1^. The Eustachian tube is vital to the pathology of this condition, as Eustachian tube dysfunction occurs in approximately 70% of individuals receiving surgical intervention for middle ear issues. Furthermore, CSOM is the leading cause of permanent hearing loss among pediatric patients in developing countries^2^. Data show that macrophages significantly contribute to CSOM-mediated sensorineural hearing loss^3^. Current evidence indicates that inflammatory mediators play a pivotal role in CSOM pathogenesis, with markedly elevated levels of interleukin (IL)−6 and IL-8 in patients with CSOM and notably higher IL-1α levels in those with cholesteatoma involvement^4^. The treatment options for chronic suppurative otitis media (CSOM) remain suboptimal; current management primarily relies on long-term topical antibiotic therapy, with surgical interventions focused on addressing complications of the inherent tympanic membrane perforation.However, issues such as recurrent infections, the emergence of drug resistance, and persistent inflammatory damage remain prevalent. This underscores the urgent need for more effective and targeted therapeutic strategies^5^.

Bacterial infection is considered to be the primary cause of CSOM, with the resulting inflammation characterized by mucosal hyperplasia, effusion, and leukocyte infiltration in the middle ear. Co-infection with bacterial and viral pathogens is a more common form of CSOM^6^. The inflammatory response (IR) in CSOM involves complex gene expression patterns that regulate immune cell recruitment, cytokine production, and tissue remodeling. However, the precise molecular mechanisms and key regulatory genes involved in the IR in CSOM remain poorly understood. Recent studies have shown that inflammation significantly contributes to the progression of various chronic conditions. Understanding the genetic basis of IRs in CSOM is crucial for the development of a targeted treatment. In conclusion, while inflammation is recognized as a key contributor to CSOM pathogenesis, the precise genes regulating the IR and their molecular mechanisms in CSOM remain incompletely characterized.

In this study, Sprague-Dawley (SD) rats with strong immune competence and maintained hearing for at least 2 years were used. A rat model of CSOM was established by inducing tympanic membrane perforation followed by inoculation with Pseudomonas aeruginosa (strain PAO1).This study aimed to identify crucial immune-related genes implicated in CSOM regulation, elucidate their underlying molecular mechanisms, and predict possible therapeutic agents for CSOM by integrating bioinformatics approaches with experimental validation. It also aimed to establish a theoretical basis to enhance the clinical treatment outcomes associated with this condition.

## Results

### CSOM model in SD rats

**Fig. 1:**
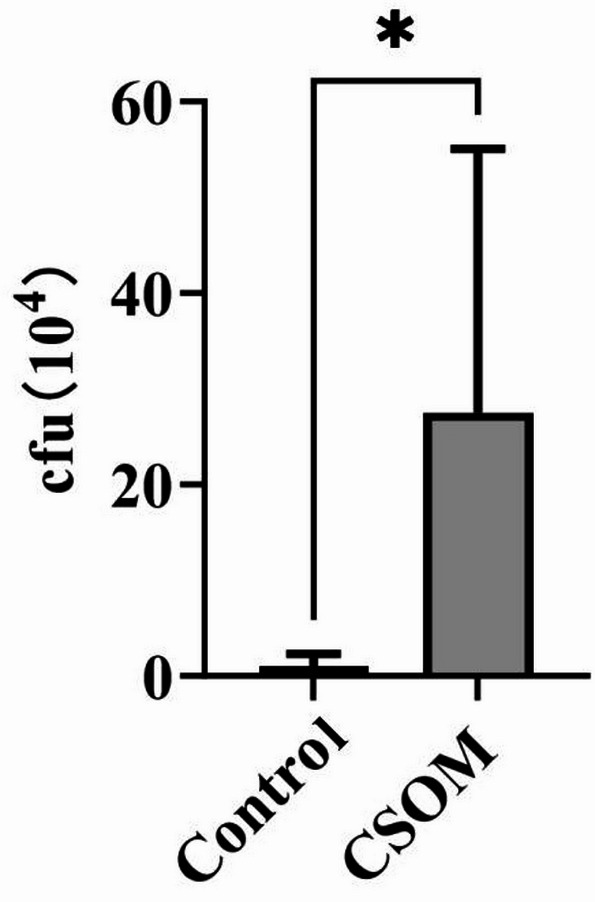
Bacterial load validation in the CSOM rat model. Colony-forming units (CFU×10^−4^) in the middle ear fluid measured 10 days following inoculation with fluorescent *Pseudomonas aeruginosa* PAO1 or sterile phosphate-buffered saline. Data are expressed as mean ± standard error of the mean (*n* = 8/group). Statistical analysis was conducted using *t*-test. *P < 0.05 versus the control group.

A CSOM model was developed to identify the key genes in CSOM. The body weights of the SD rats in the CSOM group were lower than those in the control group. The number of bacteria in the middle ear fluid significantly differed between the control and CSOM groups. Specifically, *Pseudomonas aeruginosa* inoculation in the CSOM group led to a significant increase in the number of colonies (Fig. 1).

### A total of 15 candidate genes related to IR

**Fig. 2:**
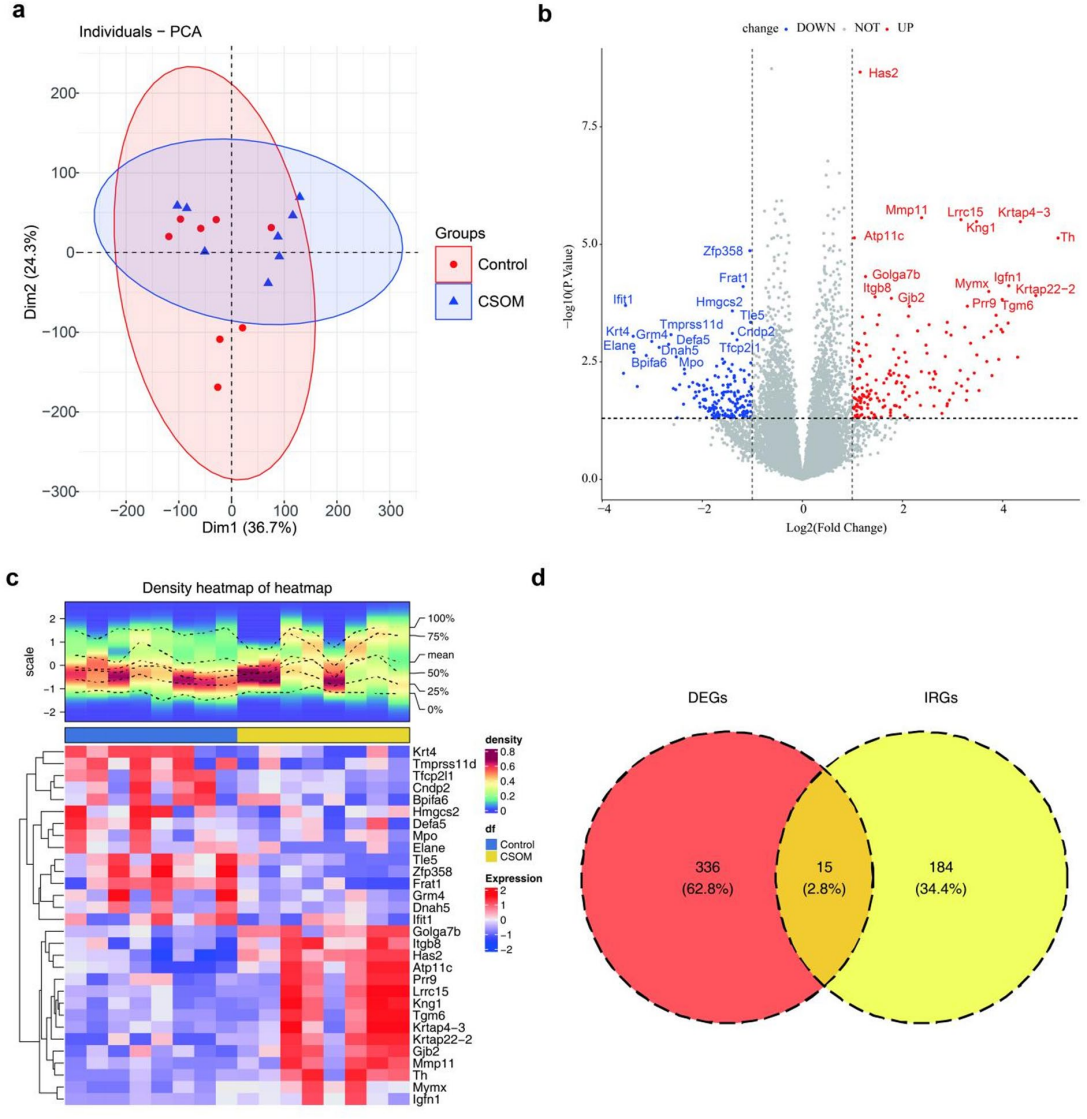
Transcriptomic analysis reveals inflammatory response-related genes in CSOM. (a) Principal component analysis conducted using the FactoMineR package demonstrating sample clustering without outliers. Each point represents one sample (*n* = 8/group). (b) Volcano plot of differentially expressed genes (DEGs) identified using the DESeq2 package. Red dots denote upregulated genes (log_2_FC > 1, *P* < 0.05); blue dots, downregulated genes (log_2_FC < − 1, *P* < 0.05); and gray dots, nonsignificant genes. The top 15 most significant up- and downregulated genes are labeled. (c) Heatmap of the top DEGs generated using the ComplexHeatmap package. Red denotes high expression, and blue indicates low expression. (d) Venn diagram showing the intersection of DEGs with inflammatory response-related genes from the MSigDB database, yielding 15 candidate genes.

Quality control was performed on the transcriptome samples, and the results indicated that the sample quality met the requirements for subsequent experimental procedures (Table 1). Principal component analysis (PCA) revealed the absence of outlier samples (Fig. 2a). Subsequently, 351 genes were identified as differentially expressed genes (DEGs) between the CSOM and control groups. Among these DEGs, 177 genes demonstrated higher expression levels in the CSOM samples, whereas 174 exhibited reduced expression levels in the CSOM samples (Fig. 2b-c). Ultimately, 15 candidate genes were obtained (Fig. 2d**)**.

**Table 1:** Basic quality control metrics for the transcriptome Samples.

Sample	Raw Data(Read)	Raw Data(Base)	Valid Data(Read)	Valid Data(Base)	Valid Ratio	Mapped Ratio	Q20	Q30	GC content
A1_Clean	45,153,445	6.77G	42,491,598	6.37G	94.10%	93.86%	99.86%	98.13%	53.50%
A2_Clean	44,651,271	6.69G	41,921,612	6.29G	93.89%	94.17%	99.45%	94.87%	53.00%
A3_Clean	37,465,425	5.62G	35,474,726	5.32G	94.69%	93.33%	99.55%	94.89%	52.50%
A4_Clean	42,587,367	6.39G	39,978,834	6.00G	93.87%	94.11%	99.87%	98.24%	53.00%
A5_Clean	42,587,367	6.39G	40,647,768	6.10G	95.45%	93.91%	99.54%	95.14%	54.00%
A6_Clean	43,624,983	6.54G	41,088,858	6.16G	94.19%	94.19%	99.87%	98.49%	56.50%
A7_Clean	44,925,879	6.74G	41,519,672	6.23G	92.42%	93.79%	99.87%	98.33%	53.00%
A8_Clean	34,995,208	5.25G	33,035,636	4.96G	94.40%	95.21%	99.86%	98.10%	53.00%
B1_Clean	36,865,991	5.53G	34,888,216	5.23G	94.64%	94.93%	99.82%	97.90%	54.50%
B2_Clean	38,057,650	5.71G	35,715,762	5.36G	93.85%	95.36%	99.77%	96.87%	55.00%
B3_Clean	41,834,027	6.28G	39,189,888	5.88G	93.68%	94.61%	99.87%	98.35%	52.50%
B4_Clean	36,734,120	5.51G	34,525,852	5.18G	93.99%	95.11%	99.85%	98.17%	54.50%
B5_Clean	35,910,732	5.39G	33,673,662	5.05G	93.77%	95.02%	99.85%	98.06%	53.00%
B6_Clean	41,267,749	6.19G	38,439,142	5.77G	93.15%	94.58%	99.74%	97.44%	50.00%
B7_Clean	44,846,046	6.73G	42,041,006	6.31G	93.75%	94.45%	99.87%	98.17%	52.50%
B8_Clean	41,050,378	6.16G	38,845,608	5.83G	94.63%	87.55%	99.84%	98.05%	55.50%

### Candidate genes exhibited a diverse range of functions

**Fig. 3:**
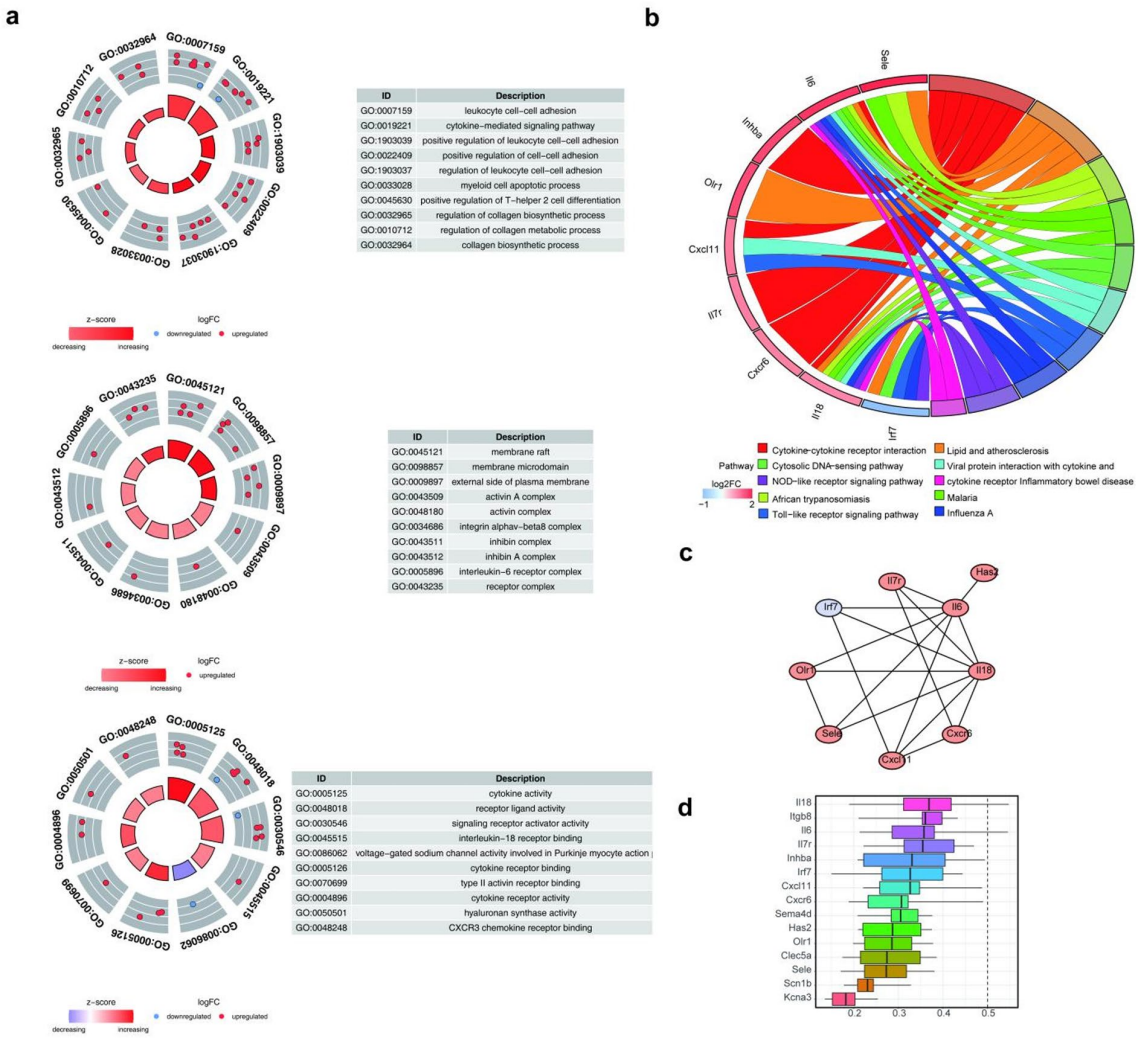
Functional characterization of candidate inflammatory genes. **(a)** Gene Ontology (GO) enrichment analysis conducted using the clusterProfiler package showing biological process, cellular component, and molecular function categories (p.adj < 0.05). **(b)** KEGG pathway enrichment analysis of candidate genes using the clusterProfiler package (p.adj < 0.05). **(c)** Protein–protein interaction network constructed using the STRING database (confidence > 0.4) and visualized in Cytoscape. Nodes represent genes, and edges represent interactions (red indicates genes upregulated in CSOM, whereas light purple denotes downregulated genes). **(d)** Functional similarity analysis of candidate genes conducted using the GOSemSim package. The color intensity represents the similarity scores.

The candidate genes were associated with different Gene Ontology (GO) categories. In particular, there were 414 entries in biological process (BP), 15 in cellular component (CC), and 25 in molecular function (MF) (adjusted *P*-value [p.adj] < 0.05) (Table S2). For example, leukocyte cell–cell adhesion, membrane raft, and cytokine activity were found to be considerably associated with the BP, CC, and MF categories, respectively (Fig. 3a). Concurrently, 20 pathways were found to be markedly associated with candidate genes (p.adj < 0.05) (Table S3). For example, cytokine–cytokine receptor interaction, lipid and atherosclerosis, African trypanosomiasis, and malaria were identified as significant pathways (Fig. 3b). Moreover, a protein–protein interaction (PPI) network of candidate genes, which contained nine nodes (representing individual genes/proteins) and 17 edges (representing interactions between genes/proteins), was developed, as illustrated in Fig. 3c. Notably, *Il6* formed a complex network with seven genes, including hyaluronan synthase 2 (*Has2*). However, the 15 candidate genes did not demonstrate a high-level functional similarity, which were all lower than 0.5 (Fig. 3d).

**Fig. 4:**
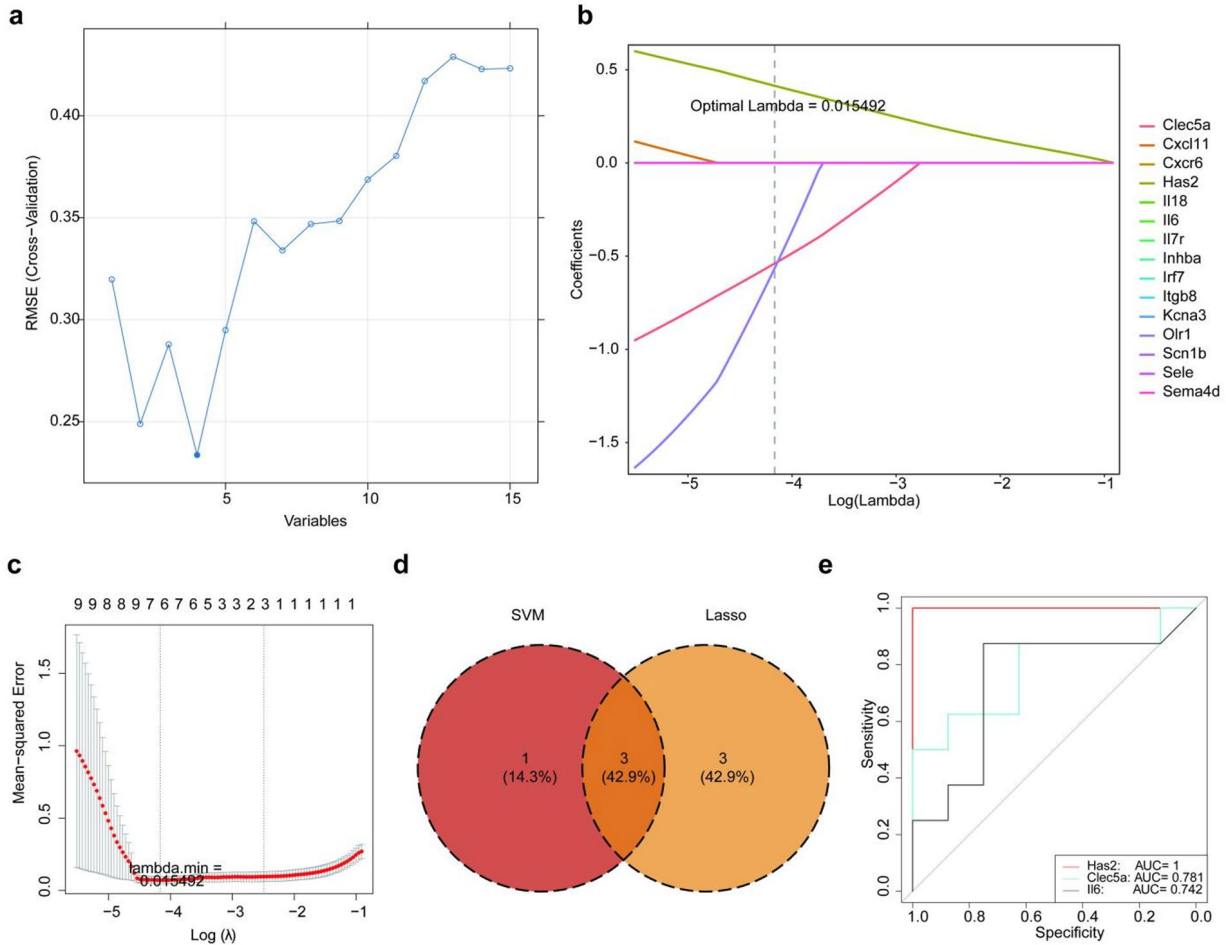
Machine learning-based identification of key biomarker genes. **(a)** Support vector machine recursive feature elimination (SVM-RFE) analysis showing cross-validation results with the optimal gene number. **(b–c)** Least absolute shrinkage and selection operator (LASSO) regression analysis conducted using the glmnet package with **(b)** coefficient paths and **(c)** cross-validation plot. **(d)** Venn diagram showing the intersection of the SVM-RFE and LASSO results, identifying 3 key genes.**(e)** Receiver operating characteristic curve analysis using the pROC package evaluating the diagnostic effectiveness of the three key genes.

A total of 15 inflammation-related candidate genes were analyzed via support vector machine recursive feature elimination (SVM-RFE), which identified the optimal subset of four key genes with the lowest prediction error (0.2336), providing the most effective feature set for CSOM classification (Fig. 4a). Concurrently, the least absolute shrinkage and selection operator (LASSO) analysis revealed an optimal six-gene model (lambda.min = 0.015492) (Fig. 4b-c). The intersection of these two gene sets yielded *Has2*, *Clec5a*, and *Il6* (Fig. 4d). This approach prevents overfitting and accurately identifies genes exhibiting the highest diagnostic value for CSOM, reducing uncertainty in subsequent experiments. In addition, the diagnostic utility of the aforementioned three genes was found to be satisfactory, as evidenced by their area under the curve (AUC) values exceeding 0.7. The respective AUC values for *Has2*, *Clec5a*, and *Il6* were 1.000, 0.781, and 0.742, respectively (Fig. 4e). Accordingly, these three genes were identified as key genes in this study.

**Fig. 5:**
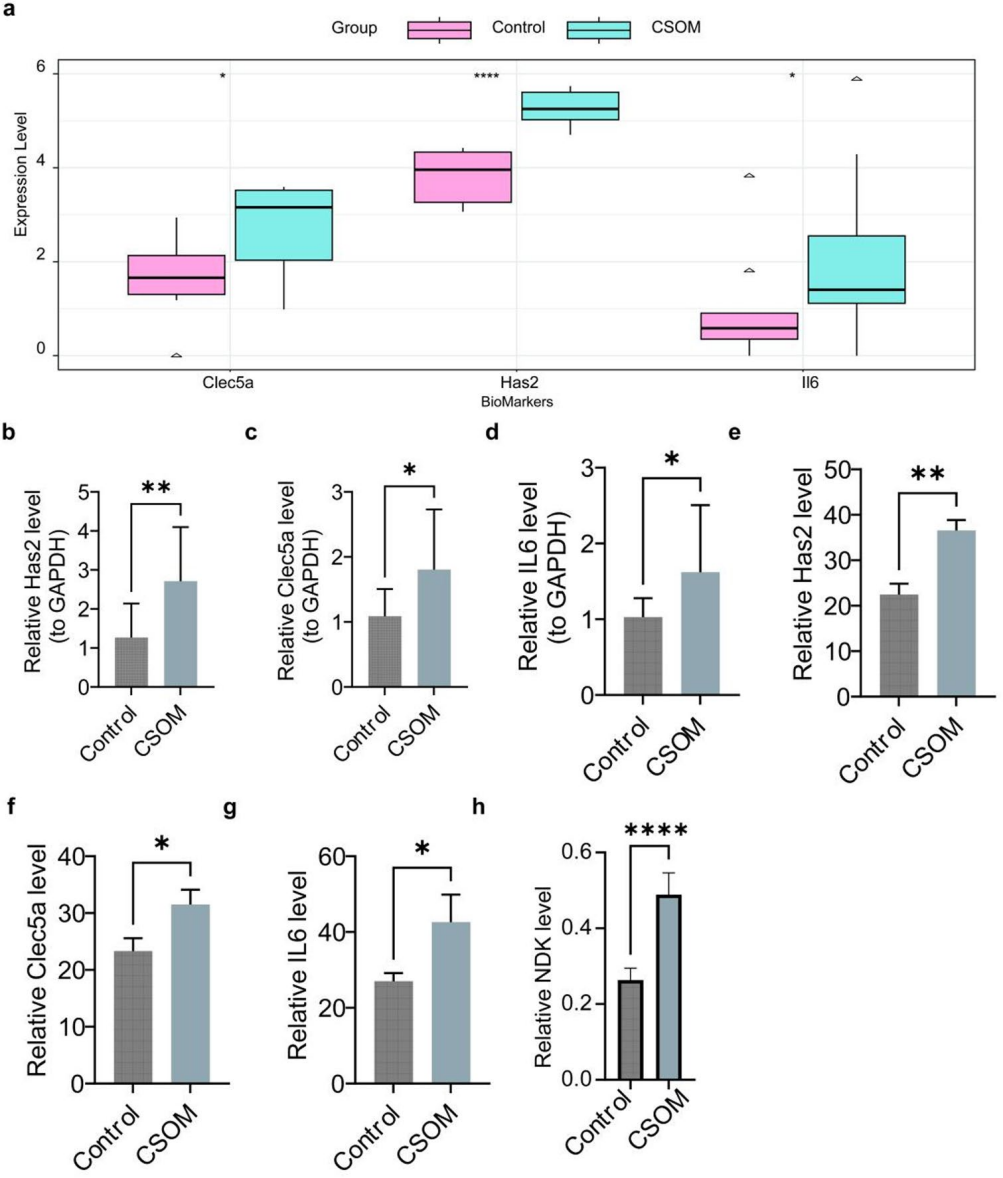
Experimental validation of key gene expression. **(a)** Expression levels of three key genes (*Has2*, *Clec5a*, and *Il6*) in RNA-seq data. Statistical comparison using the Wilcoxon test, as described in the Methods section. **(b–d)** RT-qPCR validation of (b) *Has2*, **(c)** *Clec5a*, and **(d)** *Il6* expressions. Gene expression quantified using the 2-ΔΔCt method with GAPDH as an internal control. Data are expressed as mean ± standard error of the mean (*n* = 8/group). Statistical analysis was conducted using *t*-tests. **(e–g)** Immunohistochemical (IHC) validation of **(e)** *Has2*, **(f)** *Clec5a*, and **(g)** *Il6* protein expression. **(h)** NDK protein expression analysis via IHC. Statistical analysis was conducted using *t*-tests and the GraphPad Prism software. *P < 0.05, **P < 0.01, ***P < 0.001, ****P < 0.0001 versus the control group, as described in Methods.

Interestingly, all three genes demonstrated high expression levels in the CSOM samples (Fig. 5a). Moreover, the reverse transcription quantitative PCR (RT-qPCR) results indicated that three key genes were markedly upregulated in CSOM (*P* < 0.05) (Fig. 5b-d). Moreover, consistent results were shown in the IHC results, all of which were significantly up-regulated expression (*P* < 0.05) (Fig. 5e-g). Notably, the expression of the nucleoside diphosphate kinase (NDK) protein was significantly increased in the CSOM group (*P* < 0.0001) (Fig. 5h).

### Key genes involved in variable biological effectiveness

**Fig. 6:**
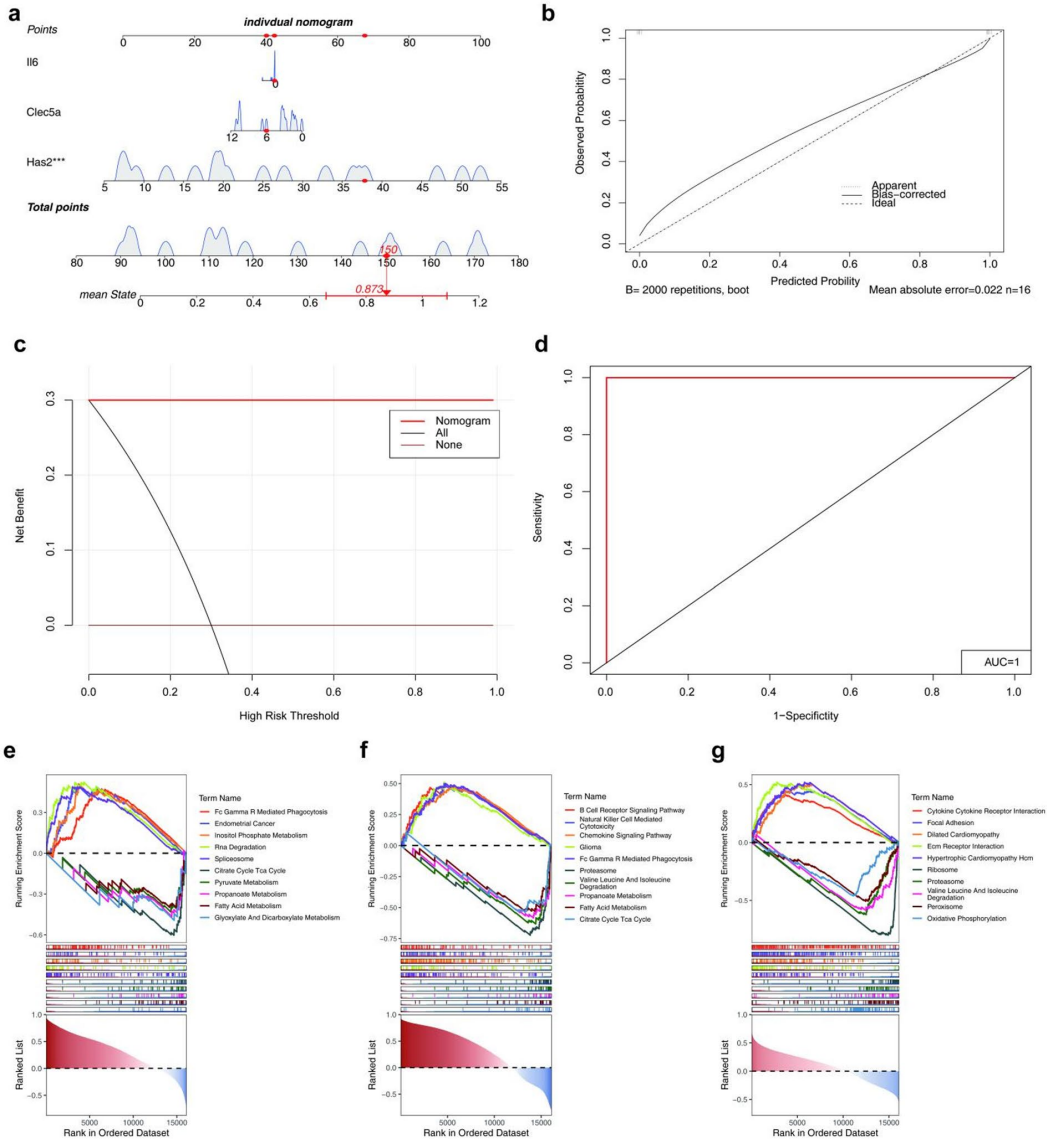
Nomogram construction and pathway enrichment analysis. **(a)** Nomogram constructed using the rms package for predicting CSOM risk based on the three key genes. **(b) **Calibration curve evaluated using the rms package, showing agreement between the predicted and actual CSOM onset. **(c)** Decision curve analysis (DCA) conducted using the rmda package, demonstrating the clinical utility of the nomogram. **(d)** ROC curve of the nomogram risk model generated using the pROC package. **(e–g)** Gene set enrichment analysis (GSEA) conducted using the clusterProfiler package for **(e)** *Has2*, **(f)** *Clec5a*, and **(g)** *Il6*, showing enriched KEGG pathways. Analysis criteria: p.adj < 0.05 and absolute normalized enrichment score (NES) > 1.

As shown in the nomogram, *Has2* exerted a more pronounced influence on CSOM, as evidenced by the longer line (Fig. 6a). Subsequently, the calibration curve showed that the mean error of the nomogram was 0.022, and the outcomes of the two groups (actual and error) of curves indicated that the effectiveness of the model (Fig. 6b). Meanwhile, decision curve analysis (DCA) revealed that the nomogram exhibited an enhanced net benefit, highlighting its exemplary predictive efficacy (Fig. 6c). Similarly, an AUC value of 1 for the ROC curve indicated that the nomogram demonstrated high-level prediction accuracy and reliability (Fig. 6d). Further analysis revealed that several key genes were present in varying quantities within the Kyoto Encyclopedia of Genes and Genomes (KEGG) pathways, suggesting their involvement in diverse BPs. In particular, *Has2*, *Clec5a*, and *Il6* were found to be involved in 61, 95, and 87 pathways, respectively. Interestingly, among the top five pathways with positive and negative normalized enrichment score (NES), *Clec5a* and *Il6* were involved in proteasome-related processes. Meanwhile, *Clec5a* and *Has2* were found to be involved in four different pathways, including propanoate metabolism and fatty acid metabolism (Fig. 6e-g).

### Various networks were predicted and linked to key genes

**Fig. 7:**
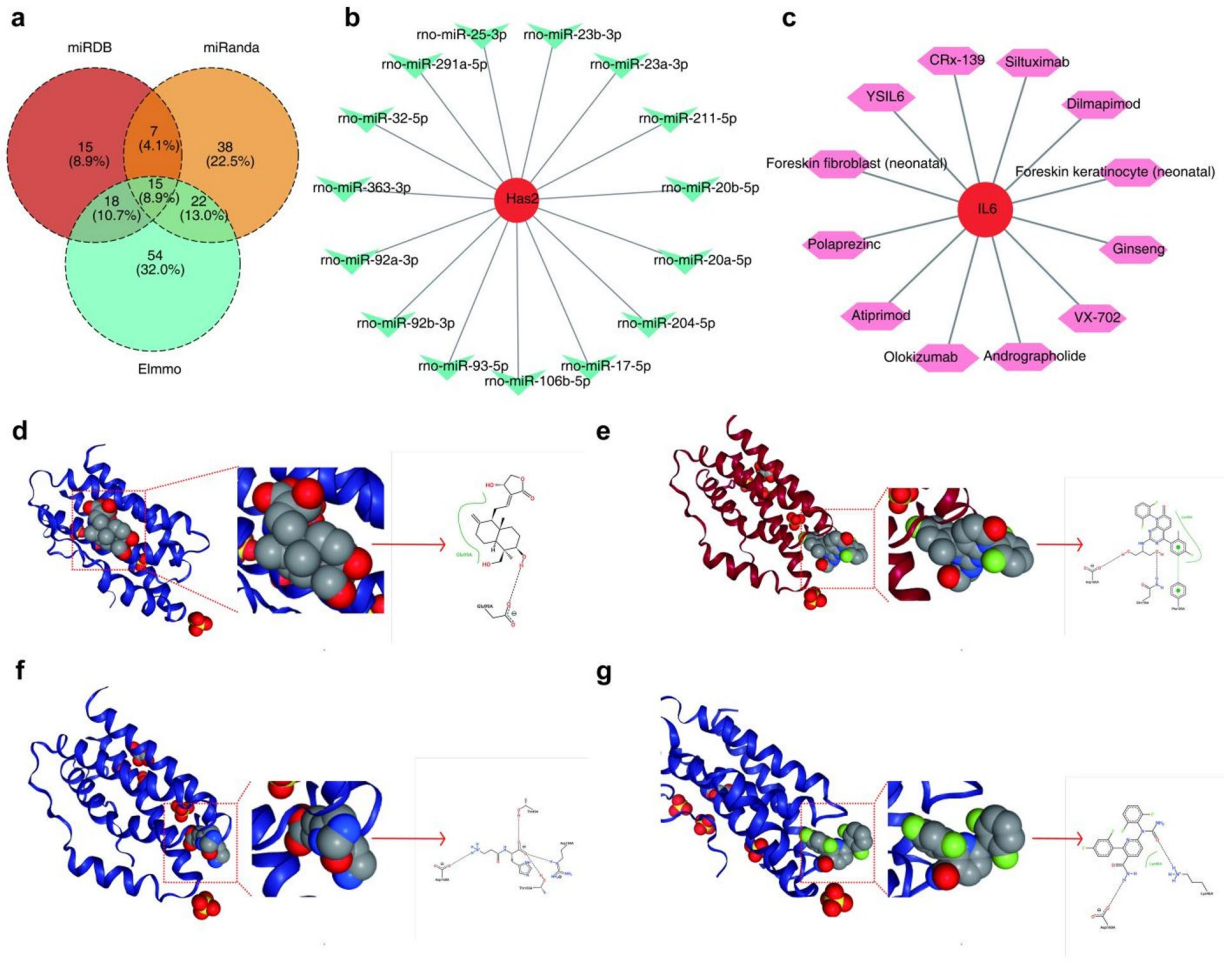
Regulatory networks and drug–target interactions. **(a–b)** miRNA–mRNA interaction networks extracted using the multiMiR package from **(a)** the miRDB, miRanda, and Elmmo databases and **(b)** intersection analysis of the three databases. **(c)** Drug–gene interaction network queried from the DrugBank database. Network visualized using Cytoscape. **(d–g)** Molecular docking analysis conducted using the AutoDock Vina platform, showing the binding interactions between *Il6* and potential therapeutic compounds: **(d)** andrographolide, **(e)** dilmapimod, **(f)** polaprezinc, and **(g)** VX-702.

*Has2* was detected as a target of 15 miRNAs, including rno-miR-32-5p, rno-miR-92b-3p, and rno-miR-92a-3p, across three databases. However, no intersecting miRNAs were identified for *Clec5a* and *Il6* (Fig. 7a-b). In addition, a drug–gene interaction analysis revealed that 12 drugs were potentially associated with the *Il6* gene. However, no drugs were found to be associated with *Has2* and *Clec5a* (Fig. 7c). Next, molecular docking was performed for six drugs in the database with a three-dimensional structure to gain insight into the potential mechanisms of drugs targeting *Il6*. These drugs include andrographolide, atiprimod, dilmapimod, polaprezinc, VX-702, and ginseng. Moreover, the docking mode among the six drugs and *Il6* showed the presence of favorable binding interactions between them; Table 2 presents in detail the specific binding energies. A docking binding energy < − 6 kcal/mol was selected for display of the docking conformation. In particular, the docking analysis revealed the presence of a hydrogen bond interaction between andrographolide and *Il6* at Glu-99 of the chain (Fig. 7d). For *Il6* and dilmapimod, two binding sites (Asp-160 and Thr-163) demonstrated hydrogen bonds, whereas the Phe-105 binding site exhibited π–π interaction (Fig. 7e). For *Il6* and polarizing, hydrogen bonds were formed at Thr-43, Asp-160, Thr-163, and Arg-104 of the chain (Fig. 7f). Hydrogen bonding interactions were observed between VX-702 and *Il6* at Lys-46 and Asp-160 on the chain (Fig. 7g).

**Table 2:** Molecular Docking parameters and binding energies of six drugs with IL6 Protein.

Target	Drug	Binding energy	center.x	center.y	center.z	size.x	size.y	size.z
IL6	Andrographolide	−6.1	0	−10	23	22	22	22
IL6	Atiprimod	−5.6	14	−31	0	24	24	24
IL6	Dilmapimod	−6.3	6	−24	18	22	22	22
IL6	Polaprezinc	−6.4	14	−31	0	19	19	19
IL6	VX-702	−6.9	14	−31	0	22	22	22
IL6	Ginseng	−4.6	14	−31	0	16	16	16

Note: Binding energies were calculated using the AutoDock Vina platform as described in the Methods section. Drug with three-dimensional structures were retrieved from the PubChem database, and the *Il6* protein structure was obtained from the Protein Data Bank (PDB). Compounds with binding energy < − 6 kcal/mol were selected for visualization of the docking conformations. Lower binding energy values indicate stronger binding affinity.

## Discussion

CSOM, sometimes referred to as chronic otitis media, is a long-lasting inflammatory and infectious disorder that affects the middle ear and mastoid cavity. This condition is characterized by ear discharge (otorrhea), which results from tympanic membrane perforation. The predominant clinical manifestations of CSOM include ear discharge and reduced auditory acuity^7^. The expression levels of inflammatory genes are positively correlated with the clinical symptoms and disease course of CSOM, indicating that these genes may serve as potential biomarkers for the early diagnosis and prognosis evaluation of the disease. Scientists aim to develop new therapeutic strategies for CSOM through in-depth research on inflammatory genes to alleviate IRs and improve patients’ hearing and quality of life^8^. Therefore, this study aimed to identify key immune-related genes involved in CSOM regulation through bioinformatics analysis and experimental validation, investigate their molecular mechanisms in CSOM pathogenesis, and predict potential therapeutic drugs for CSOM, thereby contributing to the improvement of clinical treatment outcomes for CSOM.

In this study, a differential expression analysis of the transcriptome sequencing data was conducted. This analysis enabled the identification of a set of genes that were differentially expressed between the CSOM and control groups. After intersecting with IR-related genes, IR-related DEGs were obtained. These genes mainly affect some functions related to cell epidermal adhesion and immune response, which are potential key factors in CSOM onset. Based on these genes, a PPI network was developed, and candidate biomarkers were obtained using the SVM-RFE and LASSO algorithms. Subsequently, ROC curve evaluation revealed significant expression differences among the three candidate biomarkers between the two groups and that all three biomarkers were upregulated in the CSOM group. Therefore, these three genes were utilized as biomarkers for subsequent research. The association between the three biomarkers and CSOM was established through multiple regression analysis. Then, the functions of the three biomarkers were investigated via gene set enrichment analysis (GSEA), and they were found to potentially contribute to abnormal immune-IRs in tissues and also affect some acidic substance metabolism and energy metabolism processes, thereby inducing the development of CSOM. Notably, some enriched pathways, such as those related to African trypanosomiasis and malaria, are not directly associated with CSOM etiology. However, they involve common inflammatory signaling mechanisms, such as immune cell activation, cytokine production, and host–pathogen interactions, which underlie chronic inflammatory diseases^9^. The enrichment of these pathways could be attributed to the conservation of inflammation-related genes across different disease contexts. Concurrently, a regulatory network of miRNAs for the biomarkers was constructed. Finally, computational drug prediction analysis revealed potential drugs that may target the *Il6* biomarker, which warrant further experimental validation for potential CSOM therapeutic applications.

*Has2* (hyaluronan synthase 2) is a key enzyme responsible for the synthesis of hyaluronan (HA), a glycosaminoglycan that plays a pivotal role in IRs and tissue remodeling^10^. During inflammatory processes, *Has2* expression is typically upregulated, resulting in increased HA production in inflamed tissues^11^. HA fragments of different molecular weights can exert opposing effects on inflammation: high-molecular-weight HA generally possesses anti-inflammatory properties, whereas low-molecular-weight HA generated during tissue injury can act as a damage-associated molecular pattern. These fragments promote IRs by activating Toll-like receptors and other pattern-recognition receptors^12^. As regards middle ear inflammation, increased *Has2* expression and subsequent HA accumulation may contribute to mucosal thickening, exudate formation, and persistent IR characteristic of CSOM^13^. However, the specific roles of *Has2* and HA metabolisms in chronic middle ear inflammation warrant further investigation to obtain sufficient understanding of their mechanistic contributions to CSOM pathogenesis.

*Clec5a* activation can trigger a series of cellular signaling cascades, resulting in the release of proinflammatory cytokines, such as tumor necrosis factor-alpha (TNF-α) and IL-6, thereby exacerbating IRs^14^. In addition, *Clec5a* synergizes with other pattern recognition receptors (e.g., TLR2) to enhance immune responses to bacterial infections, further promoting inflammation^15^ Furthermore, *Clec5a* gene knockout mice exhibit lower levels of inflammation and tissue damage following infection, which further supports the potential of *Clec5a* as a therapeutic target^16^. Studies have demonstrated that *Clec5a* is involved in the formation of neutrophil extracellular traps and the regulation of IRs during *P. aeruginosa* infection and that *Clec5a* blocking can alleviate bacterium-induced lung injury^17^; however, it has not been reported in otitis media, which is mainly infected by *P. aeruginosa*. It may become a novel therapeutic target for CSOM in future research.

Studies have found that the concentration of IL-6 in the ear cavity effusion of patients with CSOM is markedly higher than that in patients with other types of otitis media, indicating that IL-6 may play a pivotal role in the onset and progression of CSOM^18^. In addition, IL-6 upregulation may be associated with middle ear tissue damage and bone destruction, which is particularly evident in patients with CSOM^19^. In patients with CSOM, the IL-6 level is significantly elevated and positively correlated with the severity of the condition^4^. Therefore, targeting IL-6 and its signaling pathways may provide new avenues for CSOM treatment, which helps alleviate IRs and improve patients’ clinical outcomes. The RT-qPCR results validated that the three key genes (*Has2*, *Clec5a*, and *Il6*) identified via RNA-seq were significantly upregulated in the CSOM model, with the results highly consistent with the transcriptome data. This further enhances the reliability of the screening of key genes. As an important method for quantitative expression analysis, RT-qPCR not only confirms the accuracy of high-throughput data but also provides experimental evidence for functional studies of key genes^20^. All three aforementioned genes are closely associated with IRs, and their high expression in CSOM tissues indicates that they may be involved in the maintenance and amplification of inflammatory processes through pathways, such as immune cell activity regulation and proinflammatory factor release. In particular, studies have confirmed that *Il6*, as a classic inflammatory factor, is significantly elevated in the middle ear secretions of patients with CSOM, indicating that it plays a core regulatory role in disease progression^4^. This finding provides theoretical support for the in-depth exploration of the molecular mechanisms of CSOM and the development of new diagnostic or intervention targets.

The GSEA results indicate that the effects of the three key genes in CSOM are mainly related to the enriched pathways. For example, *Clec5a* and *Il6* are enriched in the proteasome pathway, whereas *Clec5a* and *Has2* are enriched in propanoate metabolism, Fc gamma receptor-mediated phagocytosis, fatty acid metabolism, and the citrate cycle (TCA cycle).

Ubiquitination coupled with proteasomal degradation of DNA-associated nuclear factor kappa-light-chain-enhancer of activated B cells (NF-κB), particularly its RelA subunit, is a vital mechanism for the prompt cessation of NF-κB–driven transcription of inflammatory cytokines and chemokines. After stimulation by TNF-α, WSB1/2 proteins are recruited to the chromatin where they interact with lysine-methylated RelA in a WDR-dependent manner, facilitating K48-related ubiquitination and subsequent RelA degradation. This mechanism is crucial for the timely downregulation of specific NF-κB target genes and plays a pivotal role in modulating IRs^21^. Studies have found that proteasome is an important mediator of the proliferative responses of tumor and immune cells as it regulates NF-κB signaling. Consistent with our analysis, the enrichment analysis revealed that the *Clec5a* and *Il6* genes were enriched in the proteasome pathway, indicating that these genes may regulate the occurrence of CSOM by participating in the proteasome pathway^22^. Another study showed that HRG increased the expression of Fc γ receptors (Fc γ R) and phagocytic function. Consistent with our analysis, the enrichment analysis revealed that *Clec5a* and *Has2* were enriched in the Fc gamma receptor-mediated phagocytosis pathway, indicating that these genes may regulate the occurrence of CSOM by participating in this pathway^23^.

In addition, during the inflammatory process, fatty acid metabolites influence the intensity and duration of IRs by modulating the synthesis of cytokines. For example, excessive intake of saturated fatty acids leads to chronic inflammation, potentially through the activation of the TLR4 signaling pathway, which promotes the release of proinflammatory cytokines^24^. Contrarily, the intake of ω−3 fatty acids exerts anti-inflammatory effects, as their metabolites can inhibit the synthesis of inflammatory mediators, such as IL-1β, and promote the resolution and recovery of inflammation^25^. In addition, fatty acid metabolism further regulates the inflammatory process by influencing mechanisms such as autophagy and apoptosis^26^. The enrichment analysis showed that the *Clec5a* and *Has2* genes were associated with the fatty acid metabolism pathway, suggesting a potential connection that warrants further mechanistic investigation.

A growing body of evidence suggests that andrographolide exerts beneficial therapeutic effects on various inflammatory conditions, including liver ailments, joint disorders, respiratory illnesses, neurological complications, cardiovascular diseases, inflammatory bowel disorders, and dermal inflammatory diseases^27^. Dilmapimod, an innovative p38 mitogen–activated protein kinase inhibitor, has shown promising tolerability and may warrant further investigation in extensive clinical trials aimed at preventing acute respiratory distress syndrome and other organ injuries^28^. Polaprezinc effectively eliminates the synthesis of nitric oxide and reactive oxygen species while downregulating the expression of inducible nitric oxide synthase and cyclooxygenase-2. Moreover, it suppresses the expression of crucial proinflammatory cytokines, including IL-6, TNF-α, and IL-1β^29^. VX-702 may mitigate S-AKI severity by inhibiting proinflammatory cytokine production via the MAPK pathway while obstructing the interaction of these cytokines with their respective receptors^30^. In conclusion, the study identified four drugs targeting IL-6, namely, andrographolide, dilmapimod, polaprezinc, and VX-702, which exhibited a docking binding energy < − 6 kcal/mol, indicating their potential role in modulating CSOM progression through IL-6 gene regulation. However, it should be noted that our drug prediction and molecular docking analyses provide computational evidence for potential *Il6*-targeting compounds. Nevertheless, these findings are only preliminary predictions and warrant extensive experimental validation, including in vitro and in vivo studies, before consideration of clinical therapeutic applications^31,32^. Although molecular docking results have shown favorable binding interactions, this does not guarantee their biological efficacy or safety in CSOM treatment^33,34^. Previous studies have shown that the correlation between computationally predicted results and actual biological activity is often limited by protein flexibility, cellular uptake, metabolism, and off-target effects^35,36^.

Research has shown that NDK is involved in the modulation of various cellular signaling pathways, highlighting its crucial role in acute and chronic inflammatory processes. Recent findings have indicated that NDK effectively influences the expression of genes associated with inflammation, thereby establishing a theoretical basis for its prospective use in the treatment of inflammatory diseases. In particular, NDK has been demonstrated to decrease the expression levels of inflammation-related proteins, including Toll-like receptor 4 (TLR4), NF-κB, and TNF receptor-associated factor 6, within intestinal tissues, elucidating its potential mechanisms for IR regulation^37^. Immunohistochemical (IHC) analyses revealed elevated levels of NDK protein in CSOM, further elucidating the association between IR and CSOM onset and progression. This insight provides novel perspectives for the clinical diagnosis and management of CSOM.

Through comprehensive analysis, this study has identified three biomarkers for CSOM, which mainly affect certain immune IRs as well as some acidic substance and energy metabolism processes, which may significantly contribute to CSOM onset and development. These findings provide new reference genes for CSOM diagnosis, offer new perspectives for researching the pathogenesis and progression of the disease, and provide valuable information for subsequent treatment strategies.

However, although this study has made some progress, bioinformatics analysis relies on existing databases, which often have limited sample sizes. This limitation may limit the generalizability and accuracy of the results. Increasing the sample size is essential to confirm the reliability of these findings, followed by functional experiments and in-depth molecular mechanism studies on prognostic genes. Furthermore, more detailed clinical follow-up data will be necessary in the future to confirm the value of this prognostic model.

## Methods

### Development of the CSOM model

To construct a CSOM model, 16 6–8-week-old male SD rats were purchased from Beijing Spefo Laboratory Animal Co. (Beijing, China) (production license number: SCXK (Jing) 2019-0010; use license number: SYXK (Dian) K2022-0007). All experimental protocols were approved by the Animal Welfare and Ethics Committee of Yunnan Baishitai Biotechnology Co., Ltd. (approval no.: BST-PZ-RAT-20250226-01). The experiments were conducted in strict compliance with relevant guidelines and regulations. Furthermore, the experimental reporting met the requirements outlined in the Animal Research: Reporting of In Vivo Experiments (ARRIVE) guidelines. A total of 11 rats were housed and acclimatized for 3 days after arrival to adapt to the laboratory environment. Subsequently, the animals were randomly divided into the CSOM and control groups, each consisting eight rats. After anesthesia with isoflurane, the middle ears of eight SD rats (CSOM group) were inoculated with 1 × 10^6 colony-forming units (CFU) of fluorescent-emitting *P. aeruginosa* PAO1 (PAO1.lux or PAO1.pUCP.eGFP). Pseudomonas aeruginosa is a common pathogenic bacterium in clinical CSOM cases, and its inoculation can effectively induce chronic inflammatory responses and tympanic membrane perforation characteristics of CSOM^38^. Contrarily, an equivalent volume of sterile phosphate-buffered saline (PBS) was administered to the middle ears of the control group (eight SD rats). en days post-inoculation, the bacterial load within the middle ear was evaluated using the LagoX In Vivo Imaging System (Spectral Instruments Imaging, USA), and bacterial enumeration was performed to confirm the establishment of chronic infection. At the end of the experiment, the rats were euthanized by excessive anesthesia with 2% pentobarbital at a dose of 100 mg/kg., and middle ear tissues and fluid were harvested. The collected tissues were used for further analyses, including transcriptome sequencing (RNA-seq), RT-qPCR, and IHC staining.

### RNA-seq and preprocessing

The RNA-seq method was employed to assess the middle ear tissues of the two groups. Total RNA was extracted following the TRIzol manufacturer’s protocol (Ambion, 15596018CN, USA)^39^. Then, the quality of the isolated total RNA was evaluated using the Agilent 2100 Bioanalyzer (Agilent, CA, USA). Subsequently, a fragmented RNA template was used alongside oligonucleotides as primers to facilitate complementary DNA (cDNA) synthesis and amplification within a reverse transcriptase system. The resulting products were purified, and a library was developed and sequenced using the Illumina NovaSeq 6000 platform (Illumina, San Diego, CA, USA) with a paired-end 150-bp sequencing strategy^40,41^. After obtaining clean data using the FastQC software (v 0.11.9)^42^, a comparison was performed between the dataset and the rat genome (*Rattus norvegicus*, nongenename.107.gtf) using the HISAT2 program. The gene count was calculated using the featureCounts software (v 2.0.3)^43^, which generated expression matrices for the 16 samples. The resulting expression matrix generated by featureCounts was further processed to obtain the fragments per kilobase of the exon mapped expression matrix of the RNA. A PCA was conducted using the FactoMineR package (version 2.11)^44^ in conjunction with the factoextra package (version 1.0.7)^45^.

### Differential expression analysis

The DEGs between the CSOM and control samples were identified using the DESeq2 package (version 1.42.0)^46^. The selection criteria for DEGs included a significance level of *P* < 0.05 and an absolute log2 fold change (FC) exceeding 1. Subsequently, the ggpubr package (version 0.4.0)^47^ was used to generate a volcano plot to visualize the DEGs. In this plot, the 15 most significantly up- and downregulated genes, prioritized by *P*-value, were prominently displayed. In addition, a heatmap was generated to depict the expression patterns of these highlighted genes using the ComplexHeatmap package (version 2.12.1)^48^.

To identify the most informative genes related to the IR in CSOM, we applied SVM-RFE to the candidate genes obtained from the transcriptome differential expression analysis. SVM-RFE is a widely used feature selection algorithm that iteratively ranks gene importance based on the weights assigned by a trained SVM model. In each iteration, the genes with the lowest weights—indicating the least contribution to classification—were removed, and the SVM model was retrained on the remaining genes. This recursive process continued until the identification of the optimal gene subset. Cross-validation was adopted to evaluate the performance of each gene subset and calculate the prediction error. The gene set with the lowest cross-validation error was chosen as the final feature set. Ultimately, the genes were retained.

### Functional network analysis of the candidate genes

The IRRGs were identified from a dataset comprising 199 rats by using “inflammation” as the search term within the Molecular Signatures Database (MSigDB) (https://www.gsea-msigdb.org/). Differentially expressed IRRGs were identified by intersecting the DEGs with the identified IRRGs, which were subsequently designated as candidate genes. To further explore the biological functions associated with these genes, analyses using the KEGG^49,50^ and GO were conducted using the clusterProfiler package (version 4.7.1.003)^51^. The species annotation package used was org. Rn.eg.db, and a significance threshold was established at an p.adj < 0.05. Furthermore, the candidate genes were analyzed for PPI networks using data from the STRING database (https://string-db.org/) (confidence > 0.4, species of *R. norvegicus*). The resulting network was then visualized using the Cytoscape software (v 3.9.1)^52^. Moreover, the Friends analysis was conducted using the GOSemSim package (v 2.28.1) to obtain additional insight into the association between candidate genes^53^.

### Identification and validation of the key genes

Feature genes were selected for subsequent analysis by determining the overlap between two gene groups derived from the SVM-RFE and LASSO. Both methods were performed based on candidate genes, whereas the latter was executed using the glmnet package (v 4.1.4)^54^. Next, receiver operating characteristic (ROC) curves were generated to assess the diagnostic effectiveness of the feature genes using the pROC package (v 1.18.0)^55^. In this study, the key genes were identified based on their favorable effectiveness, with an area under the curve (AUC) > 0.7. Subsequently, the expression patterns of key genes between the CSOM and control groups were compared using the Wilcoxon test.

To comprehensively evaluate the expression levels of pivotal genes, RT-qPCR and IHC were employed. For the RT-qPCR analysis, total RNA from the samples was extracted using the Trizol reagent (Ambion, 15596018CN, USA)^39^, followed by synthesis of cDNA using the SureScript First-Strand cDNA Synthesis Kit (GeneCopoeia, QP056, USA). GAPDH served as the internal control gene, and the 2-ΔΔCt method^56^was employed to quantify the expressions of the key genes. A *P*-value of 0.05 was deemed statistically significant for gene expression. Table S1 presents the primer sequences. The resulting data was statistically analyzed and visualized using GraphPad Prism (version 8.0)^57^^]^ with differences between the two groups determined via *t*-tests (*P* < 0.05).

In addition, IHC was performed using a rat two-step kit (ZSGB-BIO, PV-9004, China); specifically, the tissues obtained from the CSOM model were baked at 64 °C for 1 h, followed by deparaffinization by xylene and hydration with alcohol at different concentrations. Next, citrate buffer was utilized to repair the antigen and inactivate the endogenous peroxidase activity of the tissues by 3% H_2_O_2_. Subsequently, the primary antibody was incubated with a blocking solution consisting of 5% bovine serum albumin (BSA) 30 min. Then, dilution of the primary antibody was performed using a 2% BSA, which was refrigerated at 4℃ overnight and incubated at 37 ℃ for 30 min on the second day to rewarm it and then rinsed with PBS three times. Thereafter, 100 µL of the reaction enhancement solution was incrementally added, and the mixture was incubated at 37 °C for 20 min, followed by rinsing with PBS three times. Moreover, 100 µL of enzyme-labeled sheep anti-mouse (rabbit IgG polymer) was added dropwise, incubated at 37 °C for an additional 20 min, and rinsed with PBS three times. The tissue block was subjected to 3,3’-diaminobenzidine staining, which resulted in a pronounced staining effect, after which the staining solution was rinsed with PBS three times. The slide was subsequently immersed in hematoxylin for a restaining period of 5 min and then rinsed with distilled water before being placed in hydrochloric acid alcohol solution for differentiation lasting 10–15 s. The slide was returned to tap water to restore the blue coloration for over 15 min. Ultimately, the sections were dehydrated through a series of alcohol concentrations and then cleared with xylene, followed by sealing with neutral gum. The slides were then scanned and analyzed in their entirety. Also, the expression levels of the NKD protein among the different groups were evaluated via IHC (ZSGB-BIO, pv-9000, China).

### Construction and evaluation of the nomogram

To examine the role of key genes in predicting CSOM onset, a nomogram was generated using the rms package (v 6.5.0)^58^. A calibration curve, a DCA, and an ROC curve were then plotted to evaluate predictive ability using the rms, rmda (v 1.6)^59^, and pROC packages, respectively.

### Enrichment analysis of the key genes

The Spearman correlation between the key genes and the remaining genes within the RNA sequencing dataset was computed using the corrplot package (version 0.92)^60^. Then, GSEA was conducted using the clusterProfiler package, applying a significance threshold of p.adj < 0.05 and an absolute NES > 1. The background gene set was selected using the msigdbr package (version 7.5.1)^61^, focusing on the keyword “*Rattus norvegicus*” and the KEGG category for canonical pathways. The results included the presentation of the top five pathways exhibiting the highest and lowest enrichment scores.

### Forecast of the molecular network

MicroRNAs (miRNAs) targeting the key genes were extracted from the miRDB, miRanda, and Elmmo databases within the multiMiR package (v 1.20.0)^62^, allowing the study of regulatory interactions between these miRNAs and genes. The intersection of the results from the three miRNA sets was obtained, and the constructed network was visualized using Cytoscape.

### Prediction of drugs and molecular docking

The key genes were converted to human homologues using the biomaRt package (v 2.58.0)^63^. The Drugbank database (https://go.drugbank.com/) was then searched for information on drugs targeting the key genes (org = rno). The drug–gene network was visualized using Cytoscape. Then, molecular docking was adopted to develop a computational binding model for predicting the interactions between drugs and genes. The three-dimensional structures of the drugs were retrieved from the PubChem database (https://pubchem.ncbi.nlm.nih.gov/), whereas the structural data for the receptor proteins associated with the pivotal genes were obtained from the Protein Data Bank (https://www.rcsb.org/). Molecular docking analyses were conducted using the AutoDock Vina platform (https://vina.scripps.edu/). Molecular docking was performed via AutoDock Vina (https://vina.scripps.edu/) with the Vina scoring function to simulate ligand (ligand.pdbqt)-rigid receptor (receptor.pdbqt) interactions. For different drug-gene systems, individual search grids were set: centers by coordinates (center.x, center.y, center.z), ranges by dimensions (size.x, size.y, size.z, Å), grid spacing 0.375 Å, and sampling exhaustiveness 8. Each system’s docking was repeated independently; the lowest binding free energy conformation was selected as representative, and complexes with binding energy ≤ −6.0 kcal/mol were deemed to have potential binding activity. Subsequently, the resulting docking conformations were visualized using the PyMOL software (version 4.6.0)^64^ and proteins.plus (https://proteins.plus/).

### Statistical analysis methods

The R software (v 4.2.2) was used for bioinformatics analyses. *P* < 0.05 was considered to indicate statistical significance.

## Supplementary Information

Below is the link to the electronic supplementary material.

Supplementary Material 1

Supplementary Material 2

Supplementary Material 3

## Data Availability

The datasets generated during the current study are available in [Molecular Signatures Database] at [https://www.gsea-msigdb.org/], [STRING database] at [https://string-db.org/], [Drugbank database] at [https://go.drugbank.com/], [PubChem database] at [https://pubchem.ncbi.nlm.nih.gov/], [Protein Data Bank (PDB)] at [https://www.rcsb.org/], [proteins.plus] at [https://proteins.plus/].The datasets generated during this study are available in the NCBI repository at [https://www.ncbi.nlm.nih.gov/], reference number[PRJNA1240049].
